# The role of CXCL1 in crosstalk between endocrine resistant breast cancer and fibroblast

**DOI:** 10.1007/s11033-023-09119-4

**Published:** 2024-02-23

**Authors:** Sneha Pandithar, Daniel Galke, Ahone Akume, Artem Belyakov, Dominick Lomonaco, Amirah A. Guerra, Jay Park, Olivia Reff, Kideok Jin

**Affiliations:** Department of Pharmaceutical Sciences, Albany College of Pharmacy and Health Sciences, 106 New Scotland Avenue, BRB Room 105B, Albany, NY 12208 USA

**Keywords:** Breast cancer, Endocrine resistance, CXCL1, Fibroblast

## Abstract

**Background:**

ER positive breast cancer is currently targeted using various endocrine therapies. Despite the proven therapeutic efficacy, resistance to the drug and reoccurrence of tumor appears to be a complication that many patients deal with. Molecular pathways underlying the development of resistance are being widely studied.

**Methods and results:**

In this study, using four established endocrine resistant breast cancer (ERBC) cell lines, we characterized CXCL1 as a secreted factor in crosstalk between ERBC cells and fibroblasts. Protein array revealed upregulation of CXCL1 and we confirmed the CXCL1 expression by real-time qRT-PCR and U-Plex assay. Co-culturing ERBC cells with fibroblasts enhanced the cell growth and migration compared to monoculture. The crosstalk of ERBC cells with fibroblasts significantly activates ERK/MAPK signaling pathway while reparixin, CXCR1/2 receptor inhibitor, attenuates the activity. Reparixin displayed the ERBC cell growth inhibition and the combination treatment with reparixin and CDK4/6 inhibitor (palbociclib and ribociclib) increased these inhibitory effect.

**Conclusions:**

Taken together, our study implicates CXCL1 as a critical role in ERBC growth and metastasis via crosstalk with fibroblast and cotargeting CXCR1/2 and CDK4/6 could potentially overcome endocrine resistant breast cancer.

**Supplementary Information:**

The online version contains supplementary material available at 10.1007/s11033-023-09119-4.

## Introduction

Around 70% of breast cancer tissue express estrogen receptor alpha [1]. This subset of breast cancer can be treated by endocrine therapy that exploits the cancer’s dependence on hormone signaling for survival, proliferation, and progression. Expression of this molecule allows for endocrine specific targeting of oncogenic pathways. Therapy mechanisms for estrogen receptor include aromatase inhibitors to decrease estrogen ligand, ovarian suppression to decrease estrogen circulation, inhibiting ligand-receptor interactions using selective estrogen receptor modulators (SERMs), or degrading estrogen receptor using selective estrogen receptor down regulators (SERDs) [1]. Endocrine therapy is an effective treatment with cure rates of 50% of patients and clinical benefit in those with advanced/metastatic stages [2]. However, endocrine resistance can arise and is associated with significant morbidity and mortality.

Mechanisms of endocrine resistance involve different levels of regulation of growth signaling pathways in the cell. Estrogen receptor downstream signaling pathways are interconnected with other kinase signaling pathways and crosstalk allows for amplification or reduction of signaling to bypass ER blockade, providing the cell with alternative proliferation and survival signals or regulation of ER activity and/or signaling. Post-translational modifications of the estrogen receptor can activate downstream pathways by alteration of the ER, coregulators or other machinery involved in the pathway. Due to inherent genomic instability in cancer, mutations can occur in ESR1 that may promote expression, ligand affinity or confer constitutive activation in absence of ligand [1, 2].

The tumor microenvironment (TME) is a crucial factor in determination of anti-cancer immunity and cancer survival, proliferation and progression. TME alterations to promote a more hospitable environment by recruitment of immunosuppressive cells, soluble growth factor secretion, and increased angiogenesis have been shown to be induced by chemo and radiotherapy [3, 4]. Fibroblasts play a relevant role as part of the TME and can participate in exerting pro- or anti-cancer properties to the local environment. Fibroblasts can serve to suppress tumors by promoting anticancer immunity, proinflammatory secretome, tumor inhibitory signaling and ECM barriers to invasion while Cancer associated fibroblast (CAF) can promote oncogenic growth by growth factor secretion, other contributing cytokines and chemokines, immunosuppression of T-cells, macrophages, and neutrophils and well as ECM degradation by secreted proteases for metastasis [5, 6]. Determination of the factors that lead fibroblasts to a cancer promoting or suppressing phenotype is an active area of research.

CXCL1 expression occurs in response to inflammatory signals. In cancer tissue, CXCL1 has been found to be upregulated by gene amplification, transcription by high basal NFkB activation, presence of proinflammatory cytokines promoting expression and other regulators of CXCL1 mRNA stability. CXCL1 plays a critical role in cancer include induction of cancer cell migration, lymph node metastasis, recruitment of granulocytic myeloid derived suppressor cells into the tumor niche, tumor angiogenesis and recruitment of regulatory T cells to the TME [7]. These pro-cancer properties have been shown in breast cancer, glioblastoma, prostate cancer, colon cancer, laryngeal squamous cell carcinoma [8–15]. Here, we show that CXCL1 plays a critical role in endocrine resistant breast cancer (ERBC) cell growth via. a crosstalk with fibroblast. Moreover, we found that reparixin is a potential therapeutic drug in endocrine resistant breast cancer.

## Results

### Co-culture of ERBC cells with fibroblasts enhanced the cell proliferation and migration

**Fig. 1:**
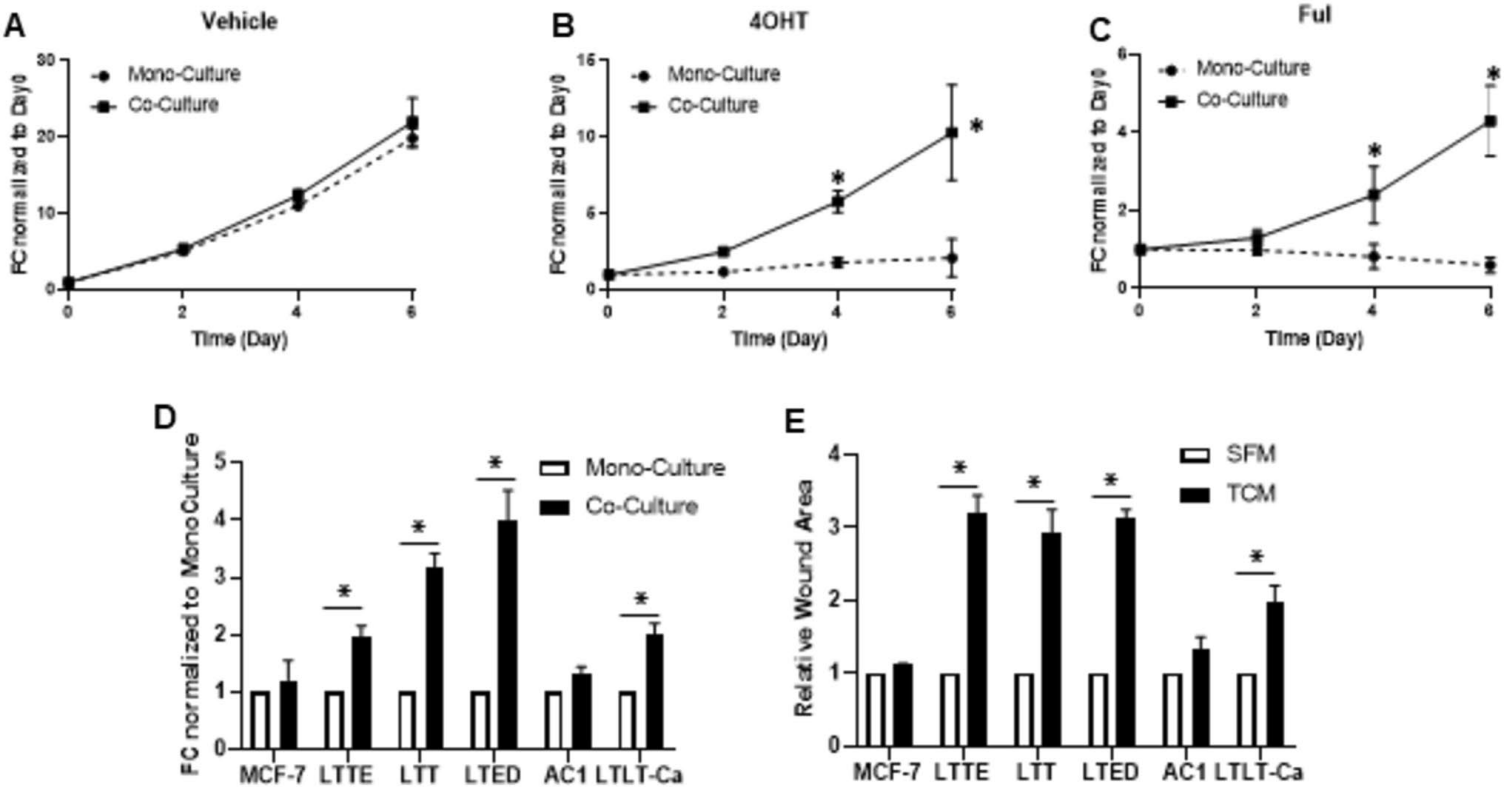
Co-culture of ERBC cells with fibroblasts enhanced the cell proliferation and migration. **A** MCF-7 cells (bottom chamber) were seeded into 96-well transwell plates with (Co-culture) or without normal fibroblasts (Monoculture) in the upper chamber, which cultured for 6 days with vehicle, **B** 1uM 4OHT, and **C** 1uM fulvestrant. Cell numbers were quantified by CyQuant Assay Kit (Life Technologies). Growth was normalized to day 0 and displayed as fold change (FC). **D** The established Endocrine Resistant Breast Cancer (ERBC) cells were co-cultured with or without normal fibroblasts and measured cell proliferation by CyQuant Assay Kit. MCF-7 parental cells and AC1 cells were used as controls. FCs induced by co-culture were obtained by normalizing to the average of monoculture controls. **E** The wound healing assays were performed to examine the migration of ERBC cells with (TCM-fibroblast) CM for 24 h and the wound area was measured by the fluorescence intensity. The serum free media (SFM) was used as a control. TCM of ERBC induced fibroblasts for 3 days and then, SFM was replaced and incubated for 24 h. The conditioned media of fibroblasts, (TCM-fibroblast) CM, were added to ERBC cells. The t-test was applied to obtain the significance. Representative data shown from three independent experiments. *P < 0.05

Cancer-associated fibroblasts (CAFs) are one of the important components in tumor microenvironment (TME) and led to drug resistance [16, 17]. In order to verify the role of fibroblasts in endocrine resistance, MCF7 cells were co-cultured with normal fibroblasts with tamoxifen and fulvestrant. Co-culturing MCF-7 cells with fibroblast promoted the MCF-7 cell proliferation with tamoxifen and fulvestrant about 5 to tenfold compared to the monocultures (Fig. 1A–C and Supplementary Fig. 1). This finding motivated the hypothesis that endocrine resistant breast cancer (ERBC) cell growth can be enhanced by co-culturing with fibroblast. We established four different endocrine resistant breast cancer (ERBC) cell lines, long-term tamoxifen treated cells in presence of estrogen (LTTE), long-term tamoxifen treated cells in absence of estrogen (LTT), and long-term estrogen deprived cells (LTED). In addition, we obtained letrozole resistant cells (LTLT-Ca) and AC1 cells as a control by Dr. Brodie. We examined the ERBC cell proliferation in the co-culture of fibroblast and found that the cell proliferation was significantly increased compared to the monocultures while MCF-7 and AC1 cells have no significance (Fig. 1D). We next investigated the ERBC cell motility in the conditioned media (CM) of fibroblasts induced by tumor conditioned media (TCM) of ERBC cells using the wound healing assays. Briefly, in order to collect the CM of fibroblast, we cultured ERBC cells confluently and harvested TCM of ERBC cells. To generate the CM of fibroblast, we cultured fibroblasts with 30% TCM of EBRC cells for three days and replacing it with the media containing 2% FBS to collect the final CM of fibroblast. After 24 h, the CM was collected and added to ERBC cells for the migration assay (Supplementary Fig. 2). The ERBC cell migration was significantly increased in the CM of fibroblasts about threefold compared to the serum free media (SFM) as a control (Fig. 1E and Supplementary Fig. 1). These results imply that the crosstalk between ERBC cells and fibroblasts enhances ERBC cell proliferation and migration.

### The expression of CXCL1 was upregulated in normal fibroblast co-cultured with ERBC cells

**Fig. 2:**
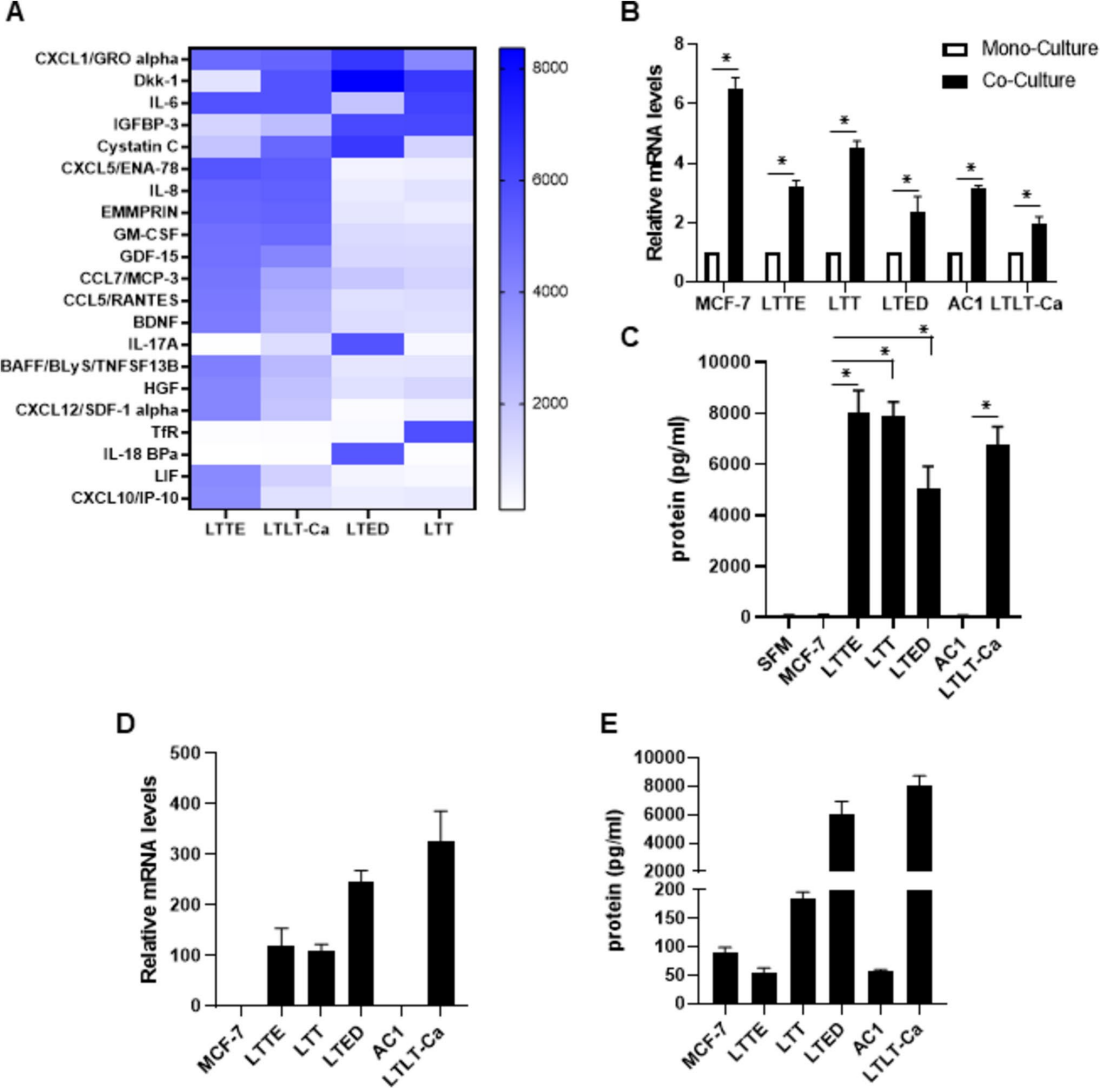
CXCL1 was upregulated in ERBC cells and fibroblast co-cultured with ERBC cells. **A** The heat maps from human cytokine antibody array (Proteome Profiler Human XL Cytokine Array Kit with 105 target proteins, R&D Systems) shows normalized fold change of 20 putative secreted factors differentially secreted from fibroblasts incubated with TCM of ERBC cells for 3 days compared to MCF-7 cells. **B** Real time qRT-PCR of CXCL1 in fibroblasts co-cultured of ERBC cells compared to single culture of fibroblasts. **C** U-Plex assay analysis of CXCL1 was performed to measure the secreted CXCL1 from fibroblasts co-cultured with ERBC cells. The serum free media (SFM) was used as a control. **D** Real time qRT-PCR of CXCL1 in ERBC cells compared to MCF-7 cells. **E** U-Plex analysis of CXCL1 was performed to measure the secreted CXCL1 from ERBC cells. The t-test was applied to obtain the significance. Representative data shown from three independent experiments. *P < 0.05

Given the observed effects of ERBC cell proliferation and migration with fibroblast, we next investigated which of the secreted factors in the conditioned media of fibroblasts induced by TCM of ERBC cells enhanced the cell proliferation and migration. We performed the reverse western assays with a human cytokine antibody array (R&D Systems) targeted for 105 human cytokines. We observed an upregulation of 10 cytokines including CXCL1, DKK-1, IL-6, IGFBP-3, Cystatin C, CXCL5, IL-8, EMMPRIN, GM-CSF, and GDF-15 using the CM of fibroblast induced by TCM of ERBC cells (fold change cut-offs of > 1.2 (Fig. 2A).

Next, we screened the 10 candidates by real-time qRT-PCR and selected CXCL1 for further study. We confirmed that the CXCL1 expression was significantly upregulated from fibroblasts included by TCM of ERBC cells using real-time qRT-PCR and U-Plex assay (Meso Scale) (Fig. 2B and C). Furthermore, we found that ERBC cells highly expressed CXCL1 compared to MCF-7 and AC1 cells (Fig. 2D and E). These results suggest that CXCL1 is a critical factor in crosstalk between ERBC cells and fibroblast through auto-and paracrine loop.

### ERK/MAPK pathway was activated in crosstalk between ERBC cells and fibroblasts

**Fig. 3:**
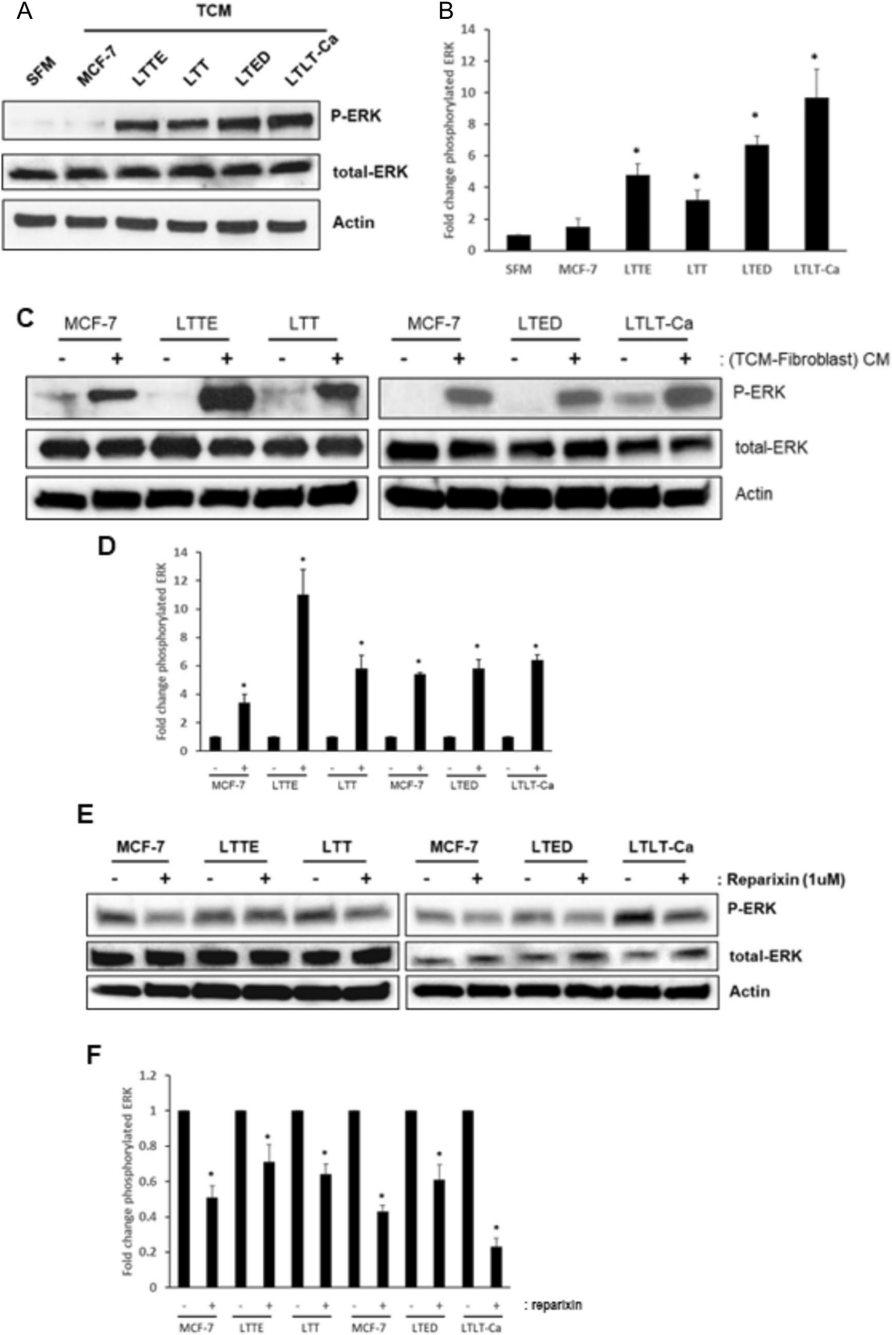
ERK/MAPK pathway was activated in crosstalk between ERBC cells and fibroblasts. **A** Fibroblasts were stimulated with TCM of ERBC for 60 min. Proteins were isolated and immunoblotting analysis of phosphorylated ERK was performed with antibodies as indicated, with β-actin as loading control. **B** The band intensities were quantified by ImageJ, and relative phosphorylation levels were calculated by correcting phosphorylation levels to total ERK protein and then normalizing to SFM controls. **C** TCM of ERBC induced fibroblasts for 3 days and then, SFM was replaced and incubated for 24 h. The conditioned media of fibroblasts, (TCM-fibroblast) CM, were added to ERBC cells for 60 min and the proteins were isolated and the immunoblots were performed and **D** the intensity of band was quantified as described above. **E** Fibroblasts were treated with 1 uM reparixin for 60 min. Proteins were isolated and immunoblotting analysis of phosphorylated ERK was performed with antibodies as indicated, with β-actin as loading control. **F** The band intensities were quantified by ImageJ, and relative phosphorylation levels were calculated by correcting phosphorylation levels to total ERK protein and then normalizing to SFM controls. Briefly, TCM of ERBC induced fibroblasts for 3 days and then, SFM was replaced and incubated for 24 h. The conditioned media of fibroblasts, (TCM-fibroblast) CM, were added to ERBC cells for 60 min and the proteins were isolated and the immunoblots were performed and the intensity of band was quantified as described above. Representative data shown from two independent experiments

Alteration of ER expression enhances tamoxifen resistance in breast cancer. The receptor tyrosine kinases induce ligand independent phosphorylation of the ER or its coactivators via activation of pAKT or MAPK [18–20]. Next, we examined the ER activity in ERBC cells by the immunoblotting analysis and found that the level of phosphorylation of ER at serine 118 was significantly increased despite the level of ER protein being widely varied (Supplementary Fig. 3A). The axis of CXCL1/CXCR1, a receptor of CXCL1, activated the MAPK kinase pathway to tumor progression [21, 22]. In order to investigate if the TCM of ERBC cells promotes the MAPK kinase signaling pathway in fibroblasts, we incubated fibroblast with TCM of ERBC cells for 1 h and examined the Phospho-ERK levels using the immunoblotting analysis and found that the phosphorylation of ERK was significantly increased compared to the SFM treatment (Fig. 3A and B). Furthermore, we incubated ERBC cells with the CM of fibroblast induced by ERBC TCM and found the upregulation of ERK phosphorylation (Fig. 3C and D). In order to confirm the effect of paracrine CXCL1 loop, we utilized reparixin, a CXCR1/2 inhibitor and incubated fibroblast with TCM of ERBC cells and found that the phosphorylation of ERK level was significantly decreased in ERBC cells with reparixin treatment compared to the vehicle control (Fig. 3E and F). The results suggest that activated paracrine loops of CXCL1 between ERBC and fibroblast play important roles in ERBC cell proliferation and migration (Supplementary Fig. 4).

### Reparixin inhibits ERBC cell grow and block the paracrine interaction between stromal and endocrine resistant breast cancer cells

**Fig. 4:**
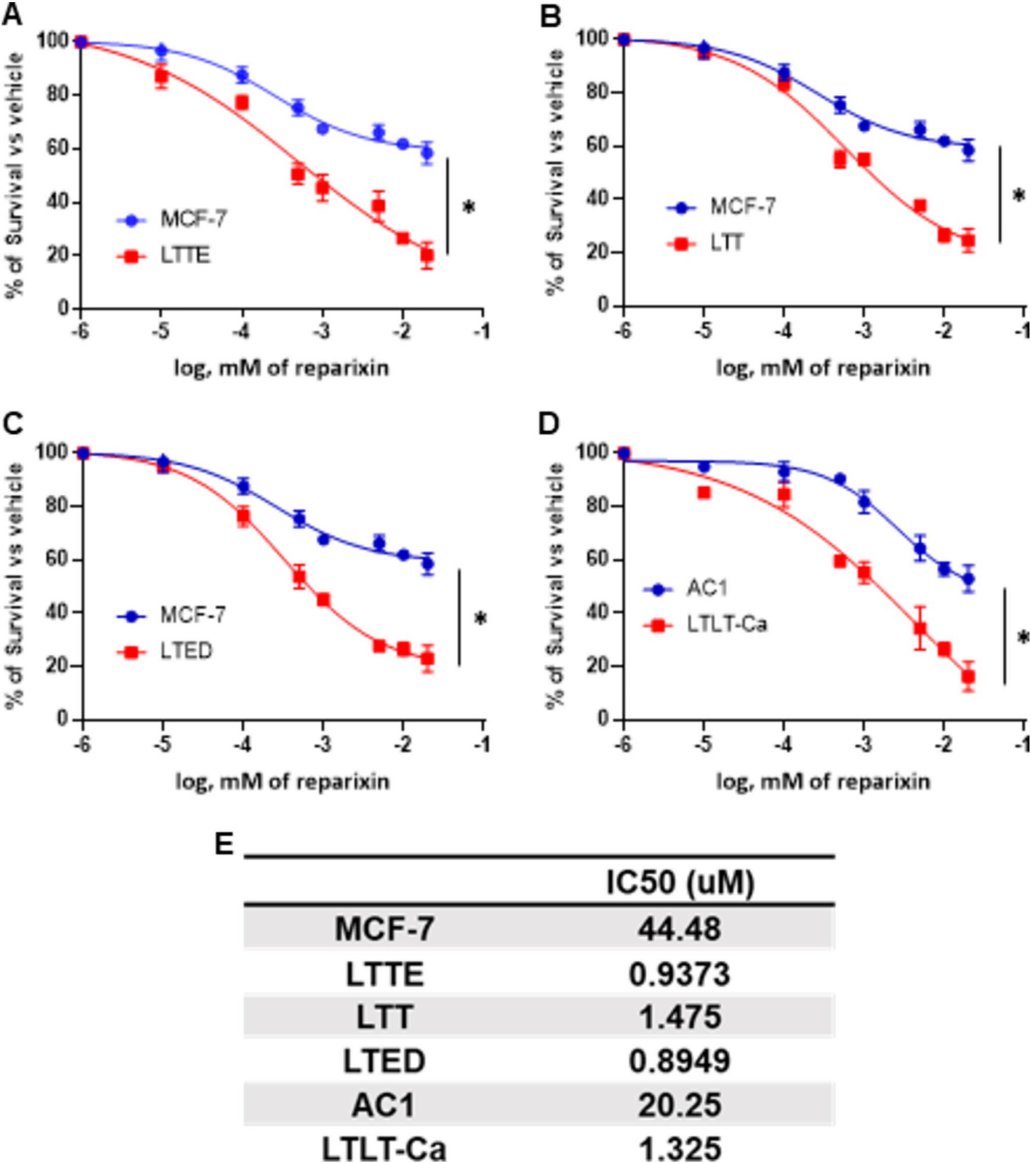
Reparixin, CXCR1/2 inhibitor, inhibited ERBC cell viability. **A**–**D** ERBC cells (bottom chamber) were seeded into 96-well transwell plates with fibroblasts (upper chamber) and cultured for 3 days with and without various concentration of reparixin. Cell numbers were quantified by CyQuant Assay Kit (Life Technologies). The percentage of cell viability was normalized to vehicle (DMSO). **E** IC50 values were determined by reparixin dose–response growth curves following the identical procedure as in **A**–**D** with reparixin concentrations as indicated. The t-test was applied to obtain the significance. Representative data shown from three independent experiments. *P < 0.05

**Fig. 5:**
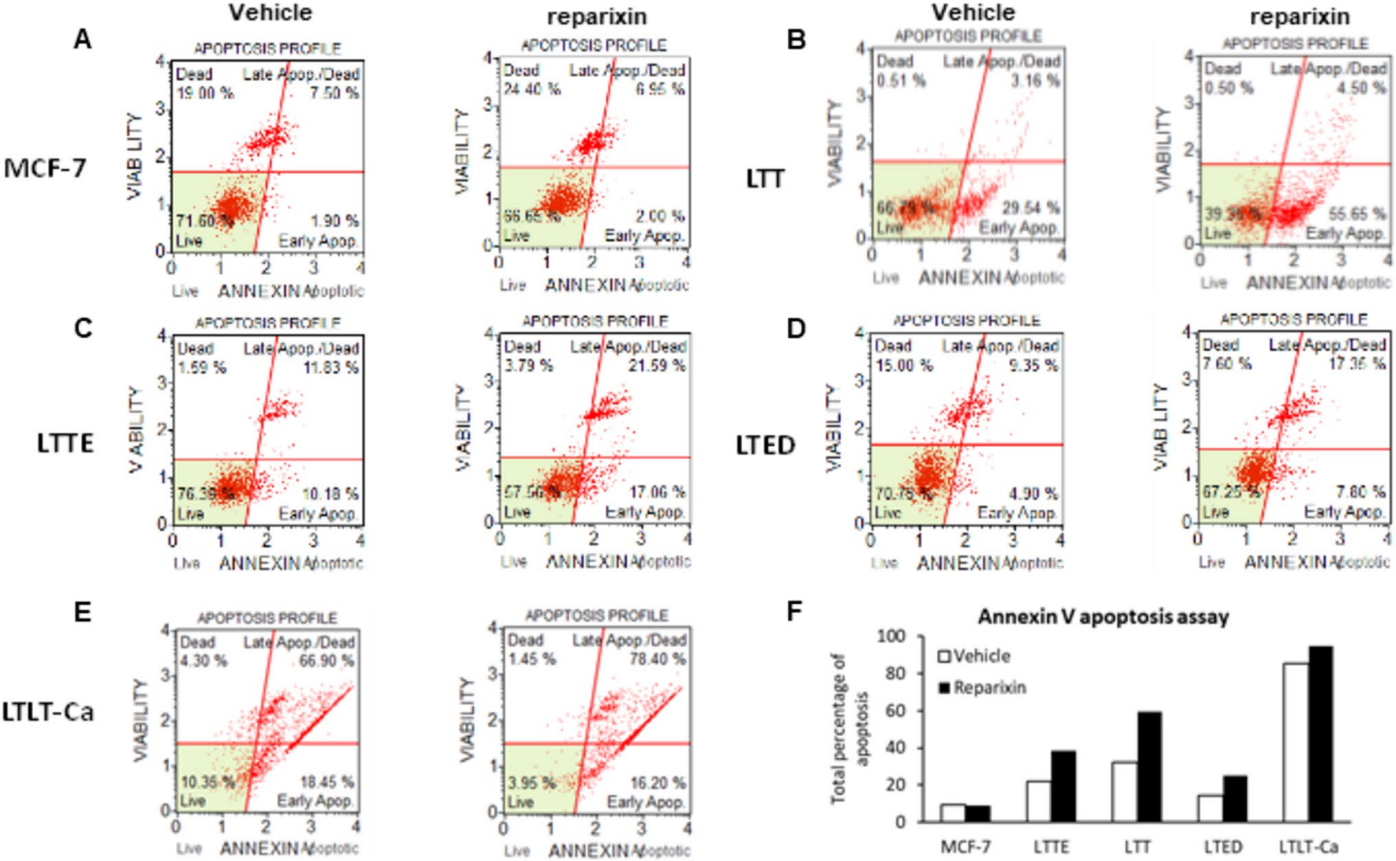
Reparixin enhanced apoptosis in ERBC cells. **A**–**E** ERBC cells were cultured with the conditioned media of fibroblasts, (TCM-fibroblast) CM and treated with 1 uM reparixin for 24 h. Cells were incubated with the Muse Annexin V & dead cell reagent (Luminex) for 20 min and measured **F** the percentage of the early and late apoptosis using Muse Cell Analyzer. Representative data shown from three independent experiments

Next, we investigated the inhibitory effect of reparixin targeting CXCR1/2 using the cell viability assay. ERBC cells were more sensitive to reparixin compared to MCF-7 and AC1 cells (Fig. 4A–D). IC50 calculated from the dose response curves suggested a significant difference between ERBC cells and control cells. ERBC cells showed a 20 to 40-fold increase in IC50 (Fig. 4E). Using the annexin V staining assay, we detected that apoptosis was significantly increased in ERBC cells treated with reparixin while the apoptosis of MCF-7 and AC1 cells was not significantly changed (Fig. 5A–F).

**Fig. 6:**
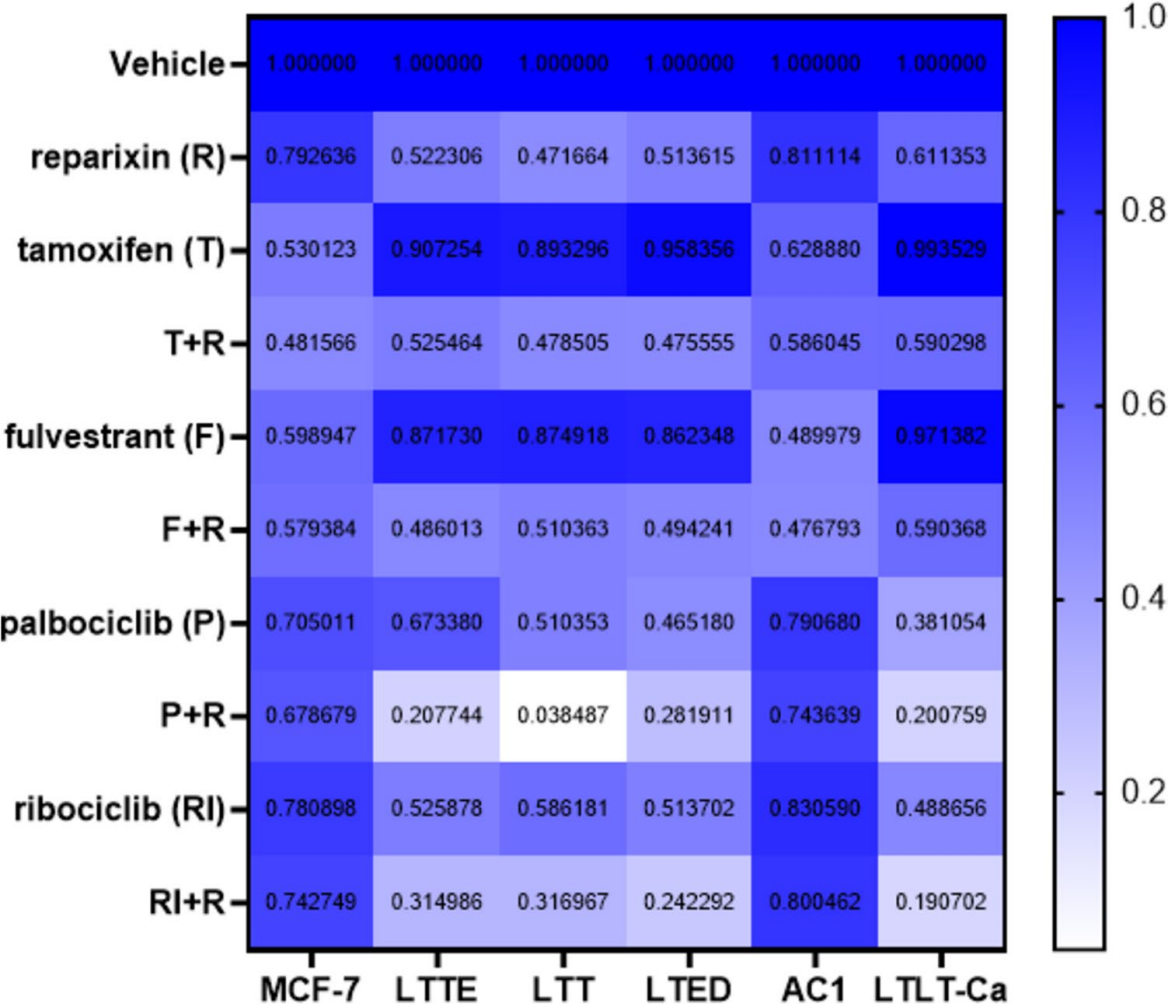
Combination treatment with reparixin and CDK4/6 inhibitors displayed synergistic effects in ERBC cells. Each ERBC cells were seeded and treated with the corresponding combined 1 uM reparixin, 1 uM 4OHT, 1 uM fulvestrant, 1 uM Palbociclib, and 1 uM ribociclib for 3 days. Cell numbers were quantified with CyQuant Assay Kit (Life Technologies). The heatmap displayed the relative cell viability calculated by normalizing to the averages of the vehicle control. The data were calculated by combining two independent experiments

Finally, we examined the combined effect of reparixin with tamoxifen, fulvestrant, and two CDK4/6 inhibitors such as palbociclib and ribociclib which are currently being used in treatment of endocrine resistant breast cancer therapy [23]. Palbociclib and ribociclib have shown the overall survival (OS) clinical benefits with AIs and fulvestrant in HR+/HER2− advanced breast cancer (ABC) patients in the studies of MONALESSA-2, MONARCH-3, PALOMA-2 [24, 25]. We treated ERBC cells with reparixin, tamoxifen, fulvestrant, palbociclib, and ribociclib by the single and combination treatment. Effects on the relative cell viability are shown in Fig. 6. The data showed that tamoxifen and fulvestrant significantly inhibited the both MCF-7 and AC1 cell viability, whereas no obvious differences were detected with reparixin, palbociclib, ribociclib, and their combination treatment. In contrast, LTTE, LTT, LTED and LTLT-Ca cells displayed the resistance to tamoxifen and fulvestrant while reparixin and the combination treatment with tamoxifen and fulvestrant enhanced the inhibitory effect. Furthermore, we found the palbociclib and ribociclib sensitized ERBC cells and observed the combined effect of reparixin with palbociclib and ribociclib. The combination of palbociclib or ribociclib with reparixin showed consistently stronger inhibitory effects compared to the combination of tamoxifen or fulvestrant with reparixin (Fig. 6 and Supplementary Fig. 1B–G). These results imply that the combination therapies cotargeting CXCL1 and CDK4/6 are a potential therapeutic strategy to inhibit endocrine resistant breast cancer.

## Discussions

With approximately 80% of the breast cancers being estrogen receptor (ER) positive, various endocrine therapies has been developed over the years that targets estrogen and estrogen receptor activity providing clinical benefits to patients consistently [26, 27]. Many studies have shown that estrogen and ER can significantly affect the therapeutic efficacy of anticancer drugs [28]. Preclinical models effective in studying molecular mechanisms by which estrogen stimulates tumor proliferation and development of endocrine resistance in ER + breast cancer have concluded that the cross talk between growth factors might be significant in development of resistance to therapy [26]. Several studies have indicated that non genomic signaling resulting in activation of ER in absence of estrogen [29].

Tumorigenesis is a complicated and multistep process that is supported largely by the tumor microenvironment. This tumor microenvironment that is composed of the endothelial cells, fibroblasts, pericytes, leukocytes and extra cellular matrix communicate with the tumor cells to support the tumor growth and metastasis. Lately, research is focused on the complex interaction between the tumor and its microenvironment that leads to proliferation and metastasis [30]. The role of tumor microenvironment in endocrine resistant breast cancer needs to be studied.

In our previous studies, we demonstrated a crosstalk between TNBC cells and lymphatic endothelial cells (LEC), fibroblasts, and macrophages [31–34]. We observed that the axis of Interleukin-6 (IL-6)/IL-6 receptor activates the JAK2-STAT3 signaling pathway, which enhances the expression of chemokine (C–C motif) ligand 5 (CCL5) in LEC. The secreted CCL5 binds to CCR5, the CCL5 receptor, which promotes TNBC tumor growth and metastasis to lymph nodes. The combination treatment of maraviroc (CCR5 inhibitor) and tocilizumab (IL-6 inhibitor) significantly block TNBC tumor growth and metastasis [34, 35]. Furthermore, we found that the axis of IL-8/CXCR1/2 plays a critical role in crosstalk between TNBC, and fibroblast and macrophage. Reparixin, CXCR1/2 inhibitor, significantly reduces TNBC tumor growth and metastasis [36].

These previous findings motivated our hypothesis that secreted factor(s) from crosstalk between endocrine resistant breast cancer and cancer associated fibroblast could be therapeutic targets to treat ERBC.

In line with this prediction, we asked if the crosstalk between ER positive breast cancer cells and fibroblasts enhanced the resistance of tamoxifen and fulvestrant, which ultimately increased the endocrine resistant breast cancer cell growth and migration. Intriguingly, we found that the co-culturing MCF-7 cells with normal fibroblast acquired the resistance to tamoxifen and fulvestrant, implicating the crosstalk between MCF-7 cells and fibroblasts confers tamoxifen and fulvestrant resistance via. secreted factors. Utilizing four established endocrine resistant breast cancer cell lines, we focused on investigating the endocrine resistant breast cancer (ERBC) growth and metastasis in the crosstalk of fibroblasts and observed the ERBC cell proliferation and migration were enhanced with co-culturing with fibroblasts.

We next asked which major secreted factors mediate the crosstalk between ERBC cells and fibroblasts. Using the protein array, we identified that CXCL1 was highly secreted in secretome from both ERBC cells and fibroblast induced by TCM of ERBC and confirmed by the real-time qRT-PCR and ELISA. In addition, we found the ERK/MAPK signaling pathway was activated by the interaction between CXCL1 and CXCR1/2 in crosstalk of ERBC cells with fibroblast while reparixin, CXCR1/2 inhibitor abolishes the ERK/MAPK activity, implicating the paracrine CXCL1 loop between ERBC and fibroblasts. Consistent with this, it was reported that the paracrine CXCL1/2 loop in endothelial-carcinoma-myeloid signaling interactions renders chemoresistance and metastasis [37].

In our search for clinically feasible approaches for inhibiting CXCL1 signaling, we utilized reparixin, which has been reported to inhibit TNBC tumor growth and metastasis [36]. Reparixin sensitized the ERBC cells and increased the percentage of apoptotic cells whereas MCF-7 cells maintained the proliferation with reparixin relatively. It could be presumably explained by the high expression of CXCL1 in ERBC cells and CXCL1 plays a critical role in ERBC cell survival. The combination of reparixin with either tamoxifen or fulvestrant has no significant inhibitory effect compared to the single dose.

As a game changer in ER positive advanced breast cancer, the cyclin-dependent kinase 4 and 6 (CDK4/6) inhibitors such as palbociclib, ribociclib, and abemaciclib, are currently treated for patients with ER positive and HER2 breast cancer [38]. Recently, many studies have sought out to the interaction between CDK4/6 inhibitors and other antitumor agents including SERM, SERD, chemotherapy agents, immune checkpoint inhibitors, autophagy inhibitor, Hippo Pathway Inhibitor, and AP-1 Inhibitor. In addition, RTK inhibitors, mTOR inhibitors, and Ras inhibitors were utilized to inhibit the CDK4/6 inhibitor resistant tumors [39]. Given the combination therapies with CDK4/6 inhibitors, this led us to hypothesize that reparixin could be a strong potential therapeutic drug partner. As expected, we observed that palbociclib and ribociclib sensitized ERBC cells. This may be explained by the high CDK4/6 activity due to the upregulation of cyclin D by highly activated ER in ERBC cells. Interestingly, we found that reparixin enhanced these inhibitor effect with both palbociclib and ribociclib about 2 to threefold.

In summary, our data indicates that ERBC cells secret CXCL1 and binds to CXCR1/2 receptor on fibroblasts, which actives ERK/MAPK pathway and upregulates CXCL1 expression. This secreted CXCL1 from fibroblasts interact with CXCR1/2 on ERBC cells and activates ERK/MAPK pathway to ERBC cell growth and migration.

Finally, these findings contribute significantly to our understanding of the role of CXCL1 as a critical factor in ERBC tumor growth and metastasis via crosstalk with fibroblast in tumor microenvironment. Furthermore, these studies suggest that the combination of reparixin and CDK4/6 inhibitors acts as the potential therapeutic regimen to circumvent endocrine resistant breast cancer growth and metastasis.

## Materials and methods

### Cell culture

MCF-7 cells were obtained from American Type Culture Collection and propagated in DMEM medium supplemented containing with 10% FBS and 1% penicillin/streptomycin (Sigma, St. Louis, MO). We established three different types of ERBC cell lines using MCF7 cells; Long-term tamoxifen treated cells in presence of estrogen (LTTE): MCF-7 cells were cultured in DMEM medium supplemented containing with 10% FBS and 1% penicillin/streptomycin with 1 uM 4-Hydroxytamoxifen (4-OHT), Long-term tamoxifen treated cells in absence of estrogen (LTT): MCF-7 cells were cultured in MEM (Richter's modification, no phenol red) medium supplemented containing with 5% CSS (charcoal stripped serum) and 1% penicillin/streptomycin with 1 uM 4-OHT, Long-term estrogen deprived cells (LTED); MCF-7 cells were cultured in MEM medium supplemented containing with 5% CSS and 1% penicillin/streptomycin. Letrozole resistant cells (LTLT-Ca) and AC1 as a parental cell line were obtained from Dr. Brodie at University of Maryland. Normal human lung fibroblasts (NHLF) were purchased from Lonza and grown in DMEM medium supplemented containing with 10% FBS and 1% penicillin/streptomycin (Sigma, St. Louis, MO). Cells were maintained under standard conditions of 37 °C and 5% CO2. Cells were cultured for a maximum of 4 weeks before thawing fresh, early passage cells and confirmed to be Mycoplasma negative (Hoechst stain).

### Conditioned media

TCM (tumor conditioned media): When ERBC cells were confluent in T175 tissue culture flasks, the normal cancer cell growth media was replaced with 8 ml serum-free media (SFM) containing with 2% serum. After 24 h incubation in a tissue culture incubator, the supernatant was centrifuged and filtered through 0.2 µm syringe filters (Corning, Corning, NY). The resulting tumor-conditioned media (TCM) was stored in aliquots at − 80 °C.

TCM-fibroblast CM (conditioned media from fibroblasts induced by TCM): When the fibroblast reached 30–40% confluence in T75 tissue culture flasks, the media was replaced with 30% of TCM of ERBC (TCM: appropriate media = 3:7) to allow the TCM to induce fibroblasts. The fibroblasts were allowed to grow in the media for 4 days at which point the media was replaced with 3 ml of SFM with 2% FBS (no supplements). After 24 h, the supernatant was centrifuged and filtered. The resulting tumor-induced fibroblast conditioned media (TCM-fibroblast) CM was stored in aliquots at − 80 °C to avoid multiple freeze thaws.

### Cell migration assay

ERBC cell migration was assessed by the would healing assay. Clear lines were created with a sterile 200 µL pipette tip. ERBC cells were continuously cultured in the growth medium for 24 h with CM of induced fibroblast or uninduced fibroblast as a control. The wound areas were recorded at 0 h and at 24 h and the wound area was quantified using a digital camera system (Cytosmart). Experiments were run in at least triplicate.

### Cell proliferation assay

The proliferation assay was measured by CyQUANT assay (Thermo fisher) according to the manufacturer’s protocol. Briefly, ERBC cells were plated at 2.5 × 10^3^ cells per well in 96-well plates, in triplicate, with 200µL (TCM-fibroblast) CM, with various inhibitors or vehicle for 3 days. 200 µL of the CyQUANT GR dye/cell-lysis buffer were added to each sample well. Mix gently and incubate the sample for 2–5 min at room temperature. Measure the sample fluorescence using a fluorescence micro­plate reader with filters appropriate for ~ 480 nm excitation and ~ 520 nm emission maxima. A percentage calculated of survival of drug-treated cells versus time-matched vehicle-treated cells.

### Antibody array

For reverse western blot, human cytokine antibody array kits (R&D Systems) were used, according to the manufacturer’s instructions. The pixel densities were analyzed using ImageJ for the experimental analysis and the Array Quick Spots Tool from HLImage++ (Western Vision Software) for the spot intensity analysis.

### UPlex assay

U-Plex Assay (multiplex ELISA) was carried out as recommended by the manufacturer (Meso Scale Discoveries, Rockville, MD). Briefly, the conditioned media samples were generated and added to a pre-coated plate containing the capture antibody per well for the detection of the CXCL1. After 3 washes, sample, standards, or calibrator were added to the wells and incubated for 1 h. Next, after 3 washes, detection antibody was added to the wells and incubated for 1 h. After a final set of 3 washes, read buffer was added to the wells and the plate was read using the MSD SECTOR Imager 2400 (Meso Scale Discoveries, Rockville, MD) and the data was processed using the MSD Workbench 4.0 software (Meso Scale Discoveries, Rockville, MD).

### Real-time qRT-PCR

Total RNA was extracted with Trizol reagent (Invitrogen), and cDNA was synthesized from total RNA (2 µg) using an iScript™ cDNA Synthesis Kits (Bio-rad). Aliquots of cDNA were used as templates for real-time RT-qPCR procedure. Relative quantitation of mRNA expression was achieved using real-time PCR (CFX96 Touch™ Real-Time PCR Detection System, Bio-rad laboratory, Hercules, California). The SYBR® Green PCR Supermixes was used according to the manufacturer’s instruction.

### Immuno blotting analysis

The western blotting analysis was performed with anti-ERK, anti-Phospho ERK (Santa cruz biotech), and anti-Actin antibody (Sigma, St. Louis, MO) as previously described [40]. Briefly, ERBC cells were incubated with (TCM-fibroblast) CM for 1 h with or without 1 uM reparixin, and fibroblasts were incubated with TCM of ERBC for 1 h. The cells were lysed with RIPA buffer, 40 ug of protein separated using a sodium dodecyl sulfate polyacrylamide gel electrophoresis gel, and then transferred onto nitrocellulose membrane. Membranes were immunostained using the above antibodies.

### Flow cytometry

To examine the apoptosis, ERBC cells were harvested following 3 days treatment with various inhibitors, or a negative control (DMSO) and were analyzed using a Muse® Annexin V Dead Cell Kit (Millipore) and the Muse® Cell Analyzer (Luminex).

### Statistical analysis

Each experiment was repeated at least three times. The results of three independent experiments performed in triplicate were shown as mean ± SD or SEM compared with control. All statistical analyses were performed using a two-sided unpaired t-test using GraphPad Prism 6 (Graphpad, La Jolla CA). A p-value of 0.05 or less was considered significant.

## Supplementary Information

Below is the link to the electronic supplementary material.

Supplementary file1 (PDF 494 kb)
