# Supplementary material for: The role of CXCL1 in crosstalk between endocrine resistant breast cancer and fibroblast
Source: Mol Biol Rep. 2024 Feb 23;51(1):331. doi: 10.1007/s11033-023-09119-4 (PMC10891235; doi:10.1007/s11033-023-09119-4)
Supplement: Supplementary file 1 — Supplementary file1 (PDF 494 kb) [file 11033_2023_9119_MOESM1_ESM.pdf]

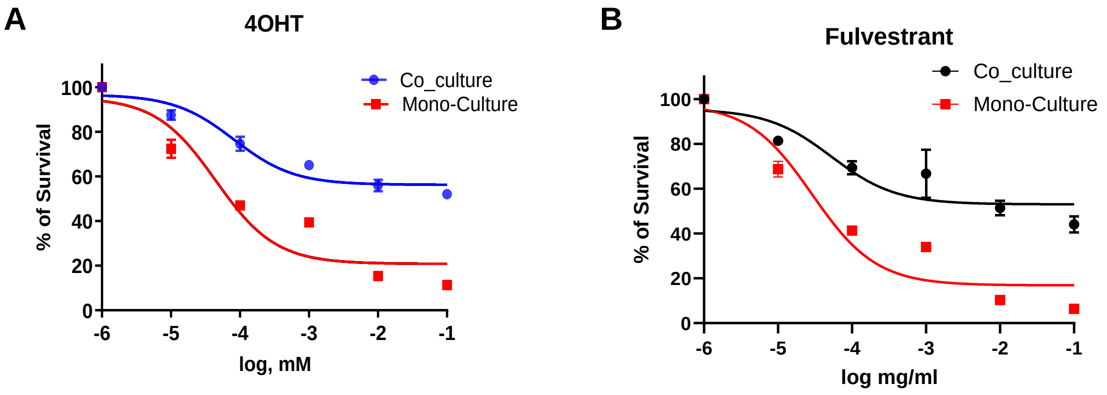

C Experimental IC50 values

	Co-culture	Monoculture
4OHT	42 uM	0.1 uM
Fulvestrant	13 uM	0.06 uM

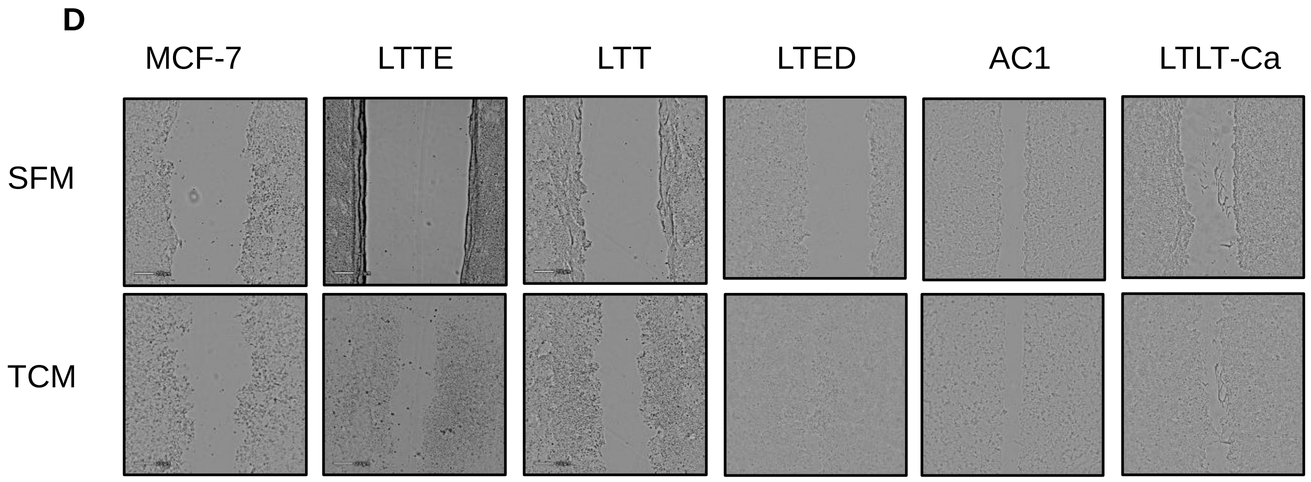

Supplementary Figure 1. (A) MCF-7 cells (bottom chamber) were seeded into 96-well transwell plates with (Co-culture) or without normal fibroblasts (Monoculture) in the upper chamber, which cultured for 3 days with various concentration of 4OHT and (B) fulvestrant. Cell numbers were quantified by CyQuant Assay Kit (Life Technologies). (C) IC50 values of MCF-7 cells treated with 4OHT and fulvestrant in mono culture or co-culture with fibroblasts. (D) The wound healing assays were performed to examine the migration of ERBC cells with (TCM-fibroblast)CM for 24 hour and the wound areas were recorded at 0 hours and at 24 hours and the wound area was quantified using a digital camera system (Cytosmart). Experiments were run in at least triplicate. Representative data shown from three independent experiments. *P<0.05.

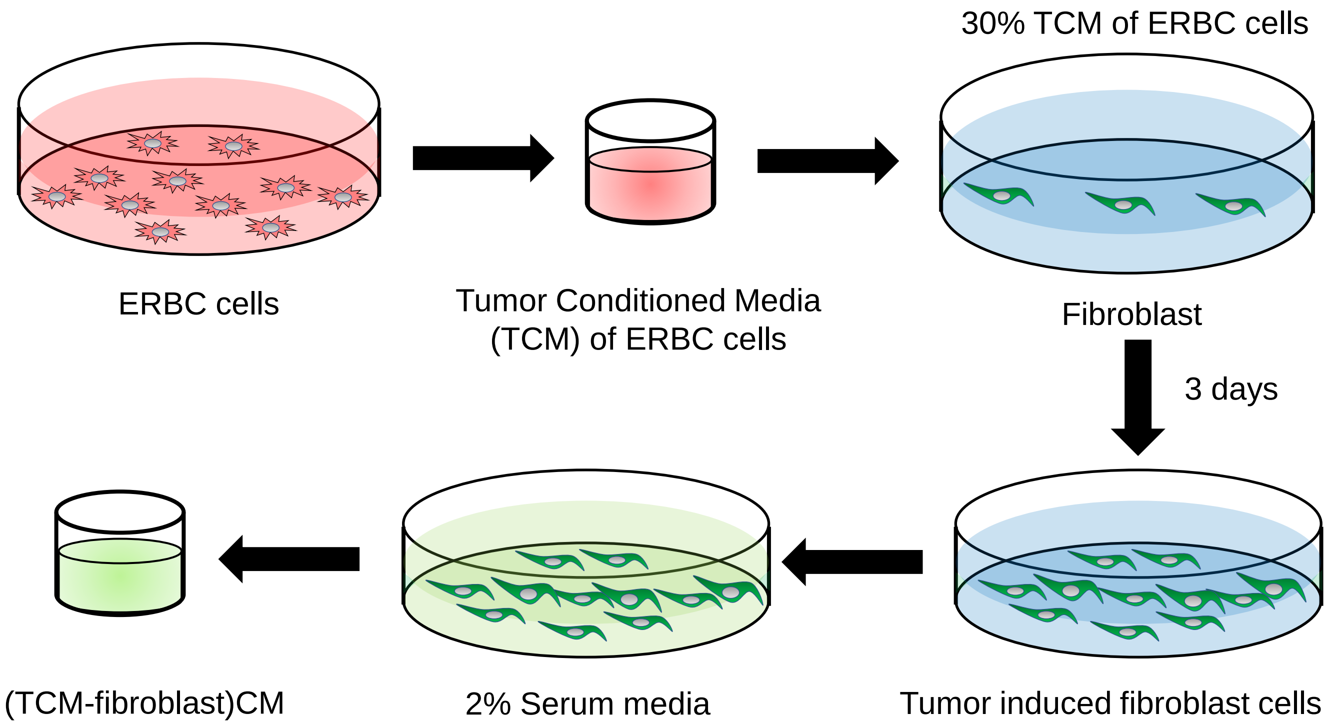

Supplementary Figure 2. Schematic diagram to generate the conditioned media of fibroblast induced by TCM of ERBC cells. Briefly, we cultured ERBC cells confluently and harvested TCM of ERBC cells. In order to collect the CM of fibroblast, we cultured fibroblasts with 30% TCM of EBRC cells for three days and replacing it with the media containing 2% FBS. After 24 hours, the final CM of fibroblast, (TCM-fibroblast)CM, was collected to use various assays.

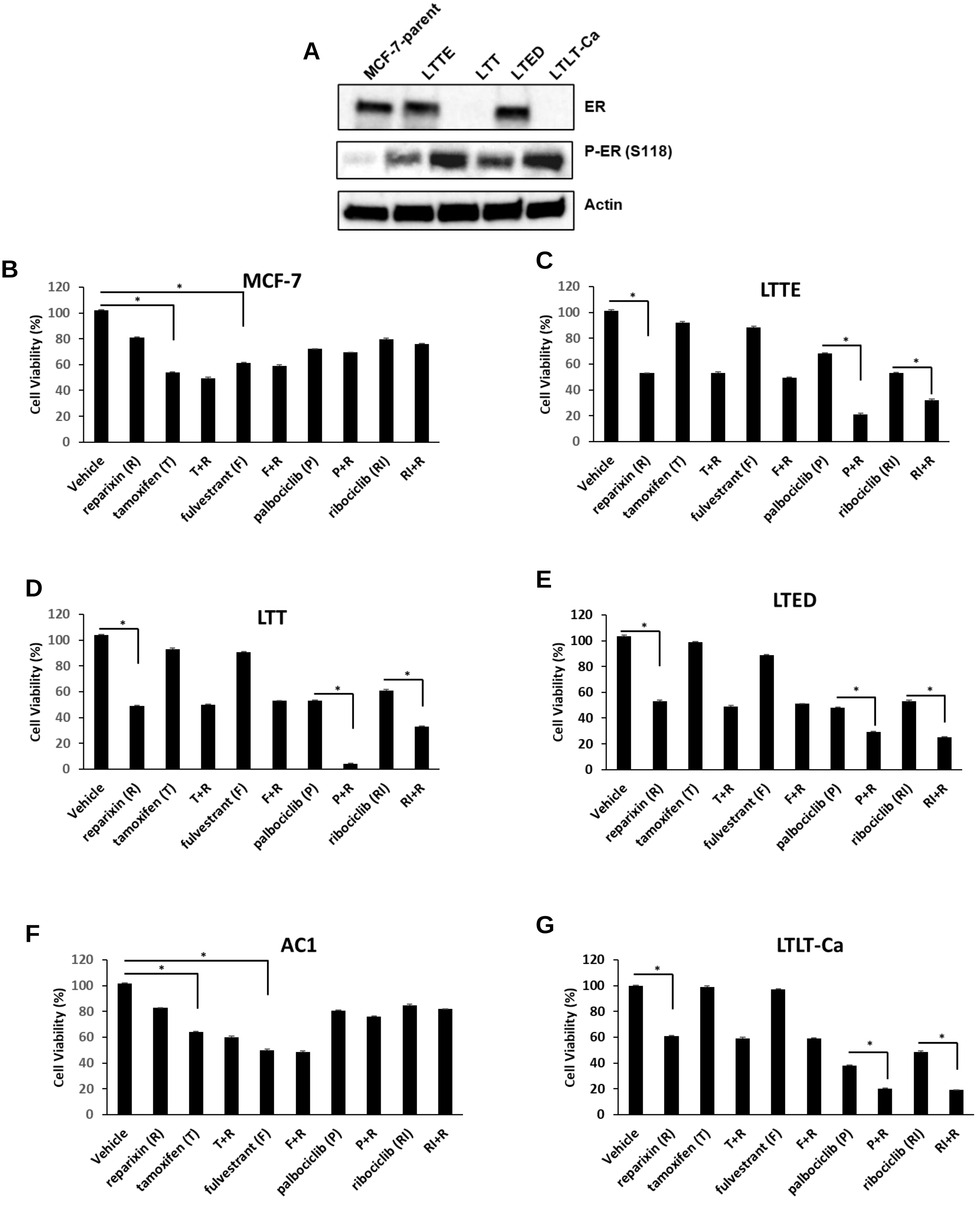

Supplementary Figure 3. (A) Immunoblotting analysis of ER and phosphorylated ER was performed and β-actin as loading control. Representative data shown from two independent experiments. (B-G Each ERBC cells were seeded and treated with the corresponding combined reparixin, 4OHT, fulvestrant, Palbociclib, and ribociclib for 3 days. Cell numbers were quantified with CyQuant Assay Kit (Life Technologies) and displayed the percentage of cell viability compared to vehicle as a control. The t-test was applied to obtain the significance. Representative data shown from three independent experiments. *P<0.05.

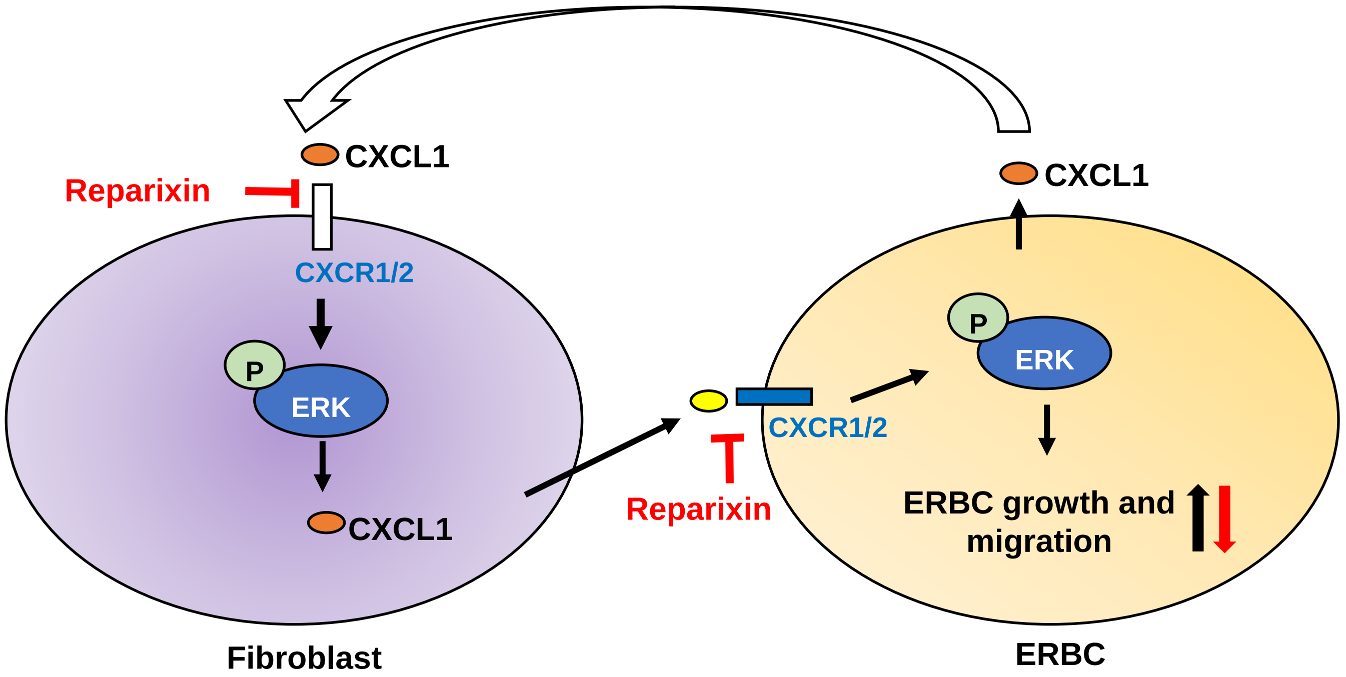

Supplementary Figure 4. The proposed model of crosstalk between ERBC cells and fibroblast. The secreted CXCL1 from ERBC cells binds to CXCR1/2 receptor in fibroblast and activate ERK signaling pathway and promotes CXCL1 expression. The secreted CXCL1 from fibroblast binds to its receptor in ERBC cells and enhances ERK pathway, which promotes ERBC cell growth and migration.
